# An equine herpesvirus type 1 (EHV-1) vector expressing Rift Valley fever virus (RVFV) Gn and Gc induces neutralizing antibodies in sheep

**DOI:** 10.1186/s12985-017-0811-8

**Published:** 2017-08-14

**Authors:** Abdelrahman Said, Mona Elmanzalawy, Guanggang Ma, Armando Mario Damiani, Nikolaus Osterrieder

**Affiliations:** 1 Institut für Virologie, Zentrum für Infektionsmedizin – Robert von Ostertag-Haus, Freie Universität Berlin, Robert-von-Ostertag-Str. 7-13, 14163 Berlin, Germany; 2 Parasitology and Animal Diseases Department, Veterinary Research Division, National Research Center, El Bouhouth St., Dokki, 12622 Cairo, Egypt; 3 Rift Valley Fever department, Veterinary Serum Vaccine Research Institute, Cairo, Egypt; 4 Instituto de Medicina y Biología Experimental de Cuyo, IMBECU-CONICET; Área de Química Biológica, Facultad de Ciencias Médicas, UNCuyo, Mendoza, Argentina

## Abstract

Rift Valley fever virus (RVFV) is an arthropod-borne bunyavirus that can cause serious and fatal disease in humans and animals. RVFV is a negative-sense RNA virus of the *Phlebovirus* genus in the *Bunyaviridae* family. The main envelope RVFV glycoproteins, Gn and Gc, are encoded on the M segment of RVFV and known inducers of protective immunity. In an attempt to develop a safe and efficacious RVF vaccine, we constructed and tested a vectored equine herpesvirus type 1 (EHV-1) vaccine that expresses RVFV Gn and Gc. The Gn and Gc genes were custom-synthesized after codon optimization and inserted into EHV-1 strain RacH genome. The rH_Gn-Gc recombinant virus grew in cultured cells with kinetics that were comparable to those of the parental virus and stably expressed Gn and Gc. Upon immunization of sheep, the natural host, neutralizing antibodies against RVFV were elicited by rH_Gn-Gc and protective titers reached to 1:320 at day 49 post immunization but not by parental EHV-1, indicating that EHV-1 is a promising vector alternative in the development of a safe marker RVFV vaccine.

## Main text

Rift Valley fever virus (RVFV) is an arthropod-borne virus that can cause serious health problems in both animals and humans [1, 2]. The disease caused by RVFV in ruminants is characterized by an acute hepatitis, abortion in pregnant animals and high mortality rates, especially in newborns [3, 4]. In humans, the virus usually leads to a mild flu-like febrile illness but in some cases, it can cause severe symptoms, such as hemorrhagic fever, hepatitis, encephalitis, and retinal degeneration [5–7]. RVFV can be transmitted from infected animals to humans, especially when humans are in contact with infected animals. Of particularly high risk are blood and aborted fetuses including the amniotic fluid and secundina [6, 8]. RVFV was first isolated from sheep during an epizootic in the Rift Valley of Kenya in 1931. RVFV is an enveloped RNA virus and belongs to the *Phlebovirus* genus in the *Bunyaviridae* family. The genome of the *Bunyaviridae* is comprised of three segments of negative-sense, single-stranded RNA that are referred to as S (small), M (medium), and L (large) with a total genome size of approximately 11.9 kb [9–11]. The M segment encodes the two major envelope surface glycoproteins Gn and Gc and two non-structural proteins NSm1 and NSm2. The Gn and Gc with molecular masses of 57- and 55-KDa, respectively [12, 13], form a heterodimer processed from a polyprotein by host proteases in the endoplasmic reticulum (ER). The glycoproteins are the main target of protective immunity against RVFV infection [14, 15]. Antibodies against surface Gn and Gc can effectively neutralize RVFV by blocking virus-receptor interactions and virus-cell entry [15]. In addition, it may also play a role in complement-mediated clearance of RVFV [13, 16]. Hence, Gn and Gc are the main targets for vaccine development [12, 13, 16–23].

Although the live attenuated [24] and inactivated vaccines [25–27] have been licensed for veterinary use, they still have some drawbacks. The ideal RVFV vaccine would be the one that (i) is safe, (ii) elicits rapid humoral immune responses that neutralize RVFV, and (iii) induces long-term protective immunity. Therefore, this study presents a different approach, using an EHV-1 strain RacH as the delivery vector. Equine herpesvirus type 1 (EHV-1) is a member of the genus Varicellovirus in the subfamily *Alphaherpesvirinae*. It possesses a double-stranded DNA genome of 150 Kbp in length. EHV-1 is capable of entering a wide variety of cell types of different origins and its attenuation could be attributed to deletion of both copies of gene 67 [28–30]. The EHV-1 vaccine strain RacH has been cloned as an infectious bacterial artificial chromosome (BAC) [31] and developed as a universal live virus vector against various viruses. RacH has a proven safety record and can induce both humoral and cellular immune responses to transgenes introduced in the vector and provide protection in vaccinated animals, including mice, dogs, cattle and swine [32–38]. In the present study, we describe the construction and evaluation of a RacH-vectored vaccine expressing Gn and Gc of RVFV (rH_Gn-Gc). We show that recombinant EHV-1 stably expresses Gn-Gc and induces a Gn-Gc-specific neutralizing antibody response in a natural host of RVFV, sheep.

**Fig. 1:**
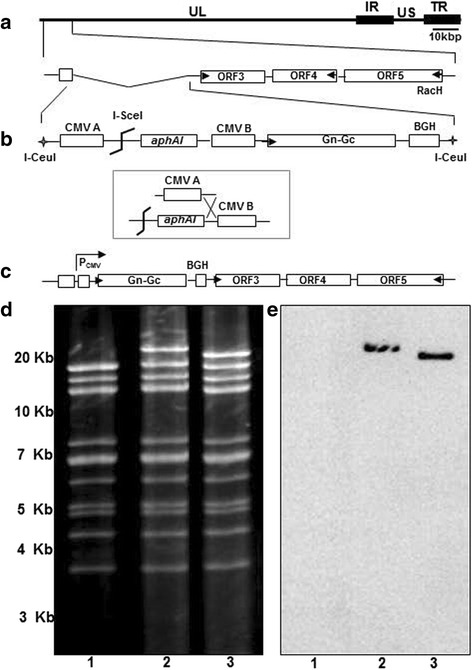
Generation of recombinant EHV-1 expressing Gn-Gc protein of RVFV (rH_Gn-Gc). Schematic illustration of the construction of rH_Gn-Gc vaccine vector based on pRacH1. **a** Depiction of the left terminus of the unique-long segment of EHV-1 strain RacH infectious BAC clone pH 1-EF1, in which ORF1 and ORF2 are naturally deleted. **b** A fragment released from transfer plasmid pUC19-ORF1/2-Gn-Gc by *I-CeuI* digestion was used to recombine with RacH genome, result in incorporation of Gn-Gc gene of RVFV, HCMV promoter and kanamycin resistance gene in the ORF1/ORF2 locus of the RacH genome. **c** After *I-SceI* digestion, kanamycin was removed in the following step of en passent mutagenesis to generate the final arrangement of rH_Gn-Gc genome. **d** and **e** Restriction fragment length polymorphisms and southern blot of pH1_EF1, the cloning intermediate and the final pH_Gn-Gc construct. An ethidium bromide-stained agarose gel is shown in the left panel with *EcoRV* restriction patterns of pH_EF1 (lanes 1), the kanamycin-resistant intermediate (lanes 2) and pH_Gn-Gc (lanes 3). GeneRuler 1 kb Plus DNA Ladder (Thermo Scientific) was used for determination of DNA fragment sizes. In the right panel, a southern blot of the same gel is shown after hybridization with a digoxigenin-labeled Gn-Gc RVFV probe

The Gn-Gc sequence of an Egyptian isolate of RVFV (ZH-501 strain; GenBank accession number DQ380200.1) was commercially synthesized after codon optimization (Genscript). Gn-Gc sequences were PCR-amplified from the commercial plasmid using Phusion high-fidelity DNA polymerase (New England Biolabs) with oligonucleotides primers P1 (TATGGATCCATGGCTGGAATTGCTATGACT) and P2 (TATGCGGCCGCTTAATTAATCTAGATTATCT) and cloned into the *BamHI/NotI* site of pEP-CMV-in [39] to generate pEP_Gn-Gc. The expression cassette containing RVF Gn-Gc under the control of HCMV IE promoter was released from pEP_Gn-Gc by digestion with *SpeI* and *SphI*, and subcloned into the *SpeI/SphI* sites of pUC19_ORF1/2, resulting in the transfer plasmid pUC19_ORF1/2-Gn-Gc. By digestion of pUC19_ORF1/2-Gn-Gc with *I-CeuI*, a 6.7 kbp fragment containing the Gn-Gc gene expression cassette, a kanamycin resistance gene (aphAI) and two flanking sequences was released and inserted in lieu of ORF1/2 of pRacH1-EF1 using two-step Red-mediated recombination (Fig. 1) as previously described [39]. The EHV-1 RacH BAC clone, pRacH1-EF1 (termed pH 1-EF1 in this study) was generated previously by replacing the HCMV IE promoter upstream of *egfp* gene in the mini-F with human elongation factor promoter 1α (EF-1α) [36, 37]. In the first recombination, insertion of Gn-Gc sequences and the aphA1 gene resulted in kanamycin-resistant intermediates that differed from parental pH 1-EF1 BAC in the *EcoRV* restriction pattern. As predicted in silico, the insertion of the cassette resulted in an *EcoRV* fragment of 21,535 bp in size compared to the 16,411 bp in the parental pH 1-EF1 (Fig. 1d). In the second recombination step, the aphA1 gene was removed, which led to the reduction in size of the 21,535 bp *EcoRV* fragment to 20,557 bp (Fig. 1d). The results of the RFLP analysis were confirmed by Southern blotting, which revealed that only the 21,535 and 20,557 bp *EcoRV* bands in the intermediate and resolved recombinant, respectively, were reactive with Gn-Gc-specific probes P3 (GCCCGATTCTTTTGTGTGCT) and P4 (AATCCGTGAAGAGGCCTGGA) (Fig. 1e). Nucleotide sequencing using oligonucleotides primers P5 (GCCGAGCGAGTTCGGCATCCT), P6 (GCCATCCTGGACCAGAACAA), P7 (GCAGGAGATCAGGAAGGCCT), P8 (CCAGCGCCATCATCGAGACC), P9 (GAGAAGCAGAAGCCCTACTT), P10 (GTGCGTGGAGAGCGAGCTGC), P11 (AGATGGAGGGCAGCCTGGCC), P12 (TCGGTCTTGGCCAGCAGCTT), P13 (GGAGCCACTGGCTCAGCTCT), P14 (GGGTGGAAGTCGGTGAAGGT), P15 (GTTCATGTCCAGCACCTCGT), P16 (CGTTGCTGCCCTTCTTGAAG), P17 (CTTGCGGTGTCGTCCTCTCC), and P18 (CTTCCGCTTGCTCTCCTCCT) further confirmed the correct insertion of the gene at the left genomic terminus of the pH 1-EF1 clone that otherwise appeared unaltered (data not shown). From the above results, we concluded that the generated recombinant pH1_Gn-Gc BAC harbored the RVFV Gn-Gc sequences in the targeted locus.

Parental RacH virus (rH) and the recombinant rH expressing Gn and Gc of RVFV (rH_Gn-Gc) were propagated in rabbit kidney (RK13) cells. Cultures were maintained in modified Eagle’s medium (MEM) (Biochrom) supplemented with 5% fetal bovine serum (FBS, Biochrom), 100 U/ml penicillin, and 100 μg/ml streptomycin (1% penicillin–streptomycin). Reconstitution of recombinant and parental viruses was achieved by transfection of pH1_Gn-Gc or pH 1-EF1 DNA into RK13 cells using polyethylenimine (PEI) (Polysciences). Reconstitution of EHV-1 gp2-encoding sequences with subsequent removal of mini-F sequences was achieved by co-transfection of 1 μg BAC DNA and 10 μg plasmid DNA p71H containing the full-length ORF71, which encodes gp2, in RK13 cells [40]. Three days after co-transfection, nonfluorescing-virus plaques were picked and purified to homogeneity by two rounds of plaque purification, and virus stocks were prepared and stored at −80 °C for further use.

**Fig. 2:**
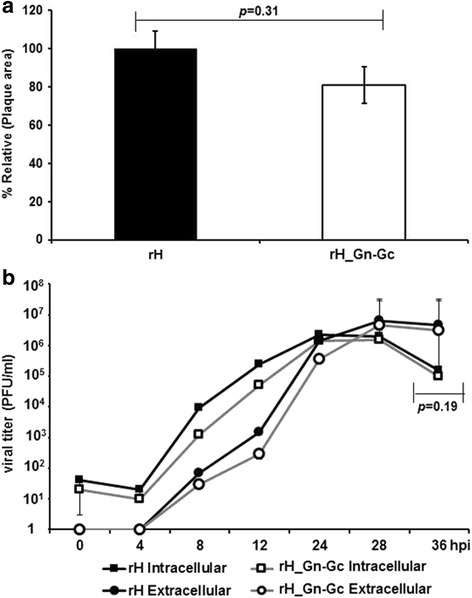
Comparison of in vitro growth properties of rH_Gn-Gc with those of parental virus. **a** RK13 cells were infected by viruses at moi of 0.001 and overlaid. Fifty plaques per virus were photographed and the areas were measured. **b** The single-step growth kinetics of those viruses was analyzed and revealed no significant differences in growth properties of parental and recombinant viruses. Error bars represent standard deviations. These results are representative of three independent experiments

To compare the in vitro growth properties of rH_Gn-Gc with those of parental rH, plaque diameters and single-step growth kinetics were determined. Plaque areas of rH_Gn-Gc were compared to those of parental rH virus, which was set as 100%. Mean percentages and standard deviations were calculated from three independent experiments. The Shapiro-Wilks test was used to assess for normality and Student’s t-test was employed to compare the mean areas of the plaques of the examined viruses. Our results shown that the average diameter of rH_Gn-Gc plaques was reduced in size by approximately 17% compared to parental virus (Fig. 2a); however, this reduction did not reach statistical significance (*p* = 0.31). To determine single-step growth kinetics, RK13 cells seeded in 12-well plates were infected at a multiplicity of infection (moi). of 3. Viruses were allowed to attach for 1 h at 4 °C, followed by a penetration step of 1.5 h at 37 °C. After washing twice with PBS, infected cells were treated with ice-cold citrate buffered saline for 3 min to remove residual virus. At different time points (0, 4, 8, 12, 24 and 36 h p.i.), supernatants and cells were harvested separately, and intracellular and extracellular viral titers were determined using plaque assay. Single-step growth curves were determined in three independent experiments and means and standard deviations were computed and plotted. Student’s t-test was used to test the differences of viral growth kinetics of examined viruses. Both viruses exhibited comparable virus titers during the 36 h observation period, with respect to both extracellular and intracellular titers (Fig. 2b). Virus titers at the end of the observation period were virtually identical between the analyzed viruses. From these results, we concluded that the insertion of transgene did not have a marked effect on viral growth in vitro.

**Fig. 3:**
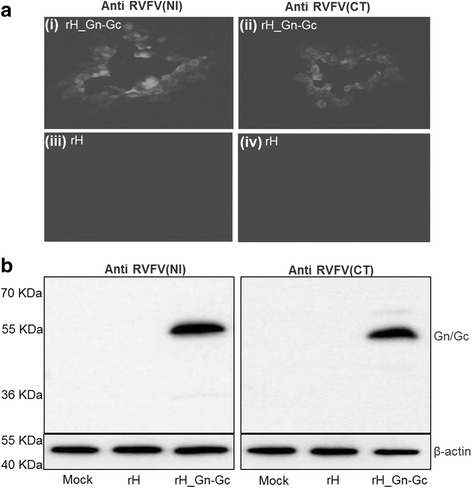
Expression of Gn-Gc protein after infection with parental rH and rH_Gn-Gc virus. **a** Immunofluorescence staining of RK13 cells infected with either parental rH or rH_Gn-Gc virus. Plaques were stained with anti-RVFV(IN) (i and ii) or anti-RVFV(CT) (iii and iv) pAb, that was reactive with RVFV Gn or Gc, respectively, followed by Alexa Flour488-conjugated goat anti-rabbit IgG. Plaques in infected cells with rH_Gn-Gc visualized by fluorescence microscopy (i and iii), whereas those induced by rH virus were not (ii and iv). **b** Cell lysates either mock infected, infected by rH or rH_Gn-Gc were separated by 10% SDS-PAGE and analysed by Western blot. Expression of Gn and Gc was detected using pAb-RVFV(IN) (left panel) and -RVFV(CT) (right panel), respectively. Expression of β-actin was determined as a loading control. The PageRulerTM Prestained protein ladder (Thermo Scientific) was used for determination of protein sizes

To evaluate expression of the Gn or Gc by rH_Gn-Gc, indirect immunofluorescence (IF) was used as described before [30, 36]. RK13 cells were infected either with rH_Gn-Gc or rH for 24 h and then incubated with rabbit anti-RVFV(CT) (ProSci catalog no. 4521) or rabbit anti-RVFV(IN) (ProSci catalog no. 4519), that recognize the RVFV Gn or Gc, respectively, for 1 h at RT. After extensive washing with PBS, the secondary antibody, anti-rabbit IgG conjugated with Alexa 488 (Invitrogen), was added at a 1:500 dilution and incubated for 30 min at RT. After thorough washing, plaques were inspected by using an inverted fluorescence microscope Zeiss Axiovert 100 and plaques recorded with the Axiocam (Zeiss). In the case of rH_Gn-Gc, virus plaques were reactive with both RVFV(IN) and RVFV(CT) pAb, whereas those induced by parental virus were not (Fig. 3a). Gn and Gc expression were also assessed by western blot analysis as described before [30, 36]. Expression of Gn and Gc was detected with the same antibodies and horseradish peroxidase-conjugated goat anti-rabbit pAb that was obtained from Southern Biotech. Expression of β-actin was assessed as a loading control using rabbit anti-β-actin polyclonal antibody (pAb) purchased from Cell Signaling Technologies. Reactive bands were visualized by enhanced chemoluminescence (ECL plus, Amersham). Proteins of approximately 57- and 55-kDa in size were reactive with the anti-RVFV(IN) and anti-RVFV(CT) antibody, respectively, in lysates of cells infected with the Gn-Gc expressing rH_Gn-Gc, but was absent in cells that were mock-infected or infected with parental virus (Fig. 3b). Our findings are in agreement with previous reports indicating that the Gn (57-KDa) and Gc (55-KDa) are produced from a single protein precursor [12, 13]. We concluded from our results that the recombinant rH_Gn-Gc efficiently expressed the RVFV Gn and Gc proteins in vitro.

**Fig. 4:**
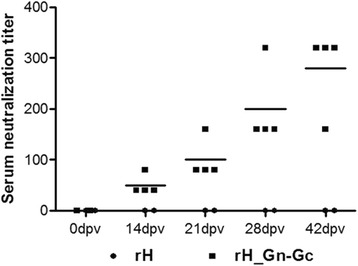
Neutralizing antibody response induced by rH_Gn_Gc. Sheep were primed and boosted with either rH or rH_Gn-Gc. Blood samples were collected from immunized sheep at indicated days 0, 14, 21, 28 and 42. Serum from immunized sheep was titrated by a standard serum neutralization test (SNT). Each dot represents an individual sheep

To test whether the rH_Gn-Gc virus could induce an RVFV specific antibody response in vivo, serological studies were done in sheep to determine whether rH_Gn-Gc was capable of inducing neutralizing antibody responses against RVFV in the natural host. All animals were screened with an enzyme linked immunosorbent assay to test for the presence of antibodies against RVFV Gn and Gc before immunization (data not shown). All sheep used in these studies were housed in isolation rooms at the Veterinary Serum Vaccine Research Institute, Cairo, Egypt. Animal care procedures were in accordance with state animal welfare guidelines under the supervision of an ethics committee. One- to five-year-old sheep were allocated randomly to two groups, with 4 sheep in group 1 and 2 animals in group 2. In group 1, sheep were immunized twice in a 3-week interval with rH_Gn-Gc (1 × 10^5^ PFU/ml) by intramuscular (IM) inoculation. In group 2 (control group), sheep were inoculated by the same route and virus amount with parental rH virus. Serum neutralizing antibodies to RVFV were determined in serum samples, collected from both group at the indicated day post vaccination (0, 14, 21, 35 and 42) by standard serum neutralization test (SNT) as described previously [41]. For SNT, RVFV strain ZH501 isolated from a human patient during the outbreak of 1977 in Egypt and kindly provided by the Naval Medical Research Unit 3 (NAMRU-3) Cairo, Egypt, was used. The virus was propagated on baby hamster kidney 21 (BHK-21) cells at the Veterinary Serum Vaccine Research Institute, Cairo, Egypt. Serum samples were examined by SNT, which revealed that all animals immunized with rH_Gn-Gc mounted high antibody titers against RVFV (Fig. 4). As expected, all animals in the rH-immunized group did not induce any RVFV-specific antibody (Fig. 4). Reduction in plaques size of RVFV by 50% (PRNT_50_) compared with control was used to quantify titer of neutralizing antibody. Our results showed that the endpoint protection titer (50% protective titer) ranged between 1:40 to 1:80 in immunized animals after first immunization dose, while the protective titers reached to 1:320 by day 49 of immunization (Fig. 4). These titers are within the range associated with protection against RVF challenge in sheep in a previous study [42]. We concluded from our findings that the engineered EHV-1 vector expressing RVFV Gn-Gc is able to induce robust neutralizing antibody responses in immunized natural hosts for RVFV.

Currently, no RVFV vaccines for humans are commercially available, but live attenuated [24] and inactivated vaccines [25–27] have been licensed for veterinary use in endemic countries. While the live attenuated vaccine is able to induce long-lasting immunity and satisfactory protection if administrated properly, its safety has been questionable. Abortions in pregnant ewes and illness in European cattle [24] were reported, as was the potential recombination with field strains and reversion to virulence during the vaccine manufacturing process. Therefore, new approaches are necessary to develop safe and effective RVFV vaccines. Several viral recombinant vectored vaccines have been developed. Those are based on vaccinia virus [43], Newcastle disease virus [12], adenovirus [13], Venezuelan equine encephalitis virus [44] or capripoxvirus [18, 45].

In this study, we explored the feasibility of using EHV-1 as a vehicle to deliver Gn-Gc of RVFV. The potential of EHV-1 as a universal vector for immunization has been previously demonstrated, including its high packaging capacity, broad cell tropism, and the lack of pre-existing anti-vector immunity in non-equine animals [46]. As a live vector, EHV-1 strain RacH has been developed and proved useful in inducing both humoral and cellular immune responses and providing protection in a number of experimental systems and of different animals, including mice, dogs, swine and cattle [32–38]. RVFV Gn-Gc are essential and sufficient for immune protection, as reported in the previous studies using baculovirus and sheeppox expression of these same two RVFV proteins [23]. Gn-Gc sequences under the control of the HCMV IE promoter was inserted in the ORF1 locus, which encodes a protein mediating evasion of T-cell immunity [47, 48]. In line with previous studies [32–37], insertion of Gn-Gc into the EHV-1 genome did not affect in vitro growth characteristics and the recombinant virus was able to replicate in cell culture as efficiently as the parental virus and stably expressed Gn-Gc. Importantly, when we inoculated the recombinant virus into sheep, a RVFV-specific neutralizing antibody response was induced following IM administration in sheep. Antibody titers were maintained at high levels up to the time point when the experiments were terminated.

In summary, we developed a recombinant EHV-1 vaccine encoding RVFV Gn-Gc and evaluated its potential as a vaccine by measurement of RVFV-specific neutralizing antibody in sheep. Our results show that EHV-1 could be used as an alternative live vector for RVFV immunization in sheep. Future study will be designed to determine whether the recombinant EHV-1-vectored Gn-Gc vaccine is capable to protect sheep against challenge infection.

## Abbreviations

aphI: Kanamycin resistance gene
BAC: Bacterial artificial chromosome
DNA: Deoxyribonucleic acid
egfp: Enhanced green fluorescence protein
EHV-1: Equine herpesvirus type1
ER: Endoplasmic reticulum
Gc: Glycoprotein C
Gn: Glycoprotein N
HCMV: Human cytomegalovirus
IM: Intramuscular
L: Large
M: Medium
moi: Multiplicity of infection
Ns: Non-structural protein
ORF: Open reading frame
PEI: Polyethylenimine
PFU: Plaques forming unit
rH: Recombinant equine herpesvirus RacH strain
RK13: Rabbit kidney cells
RNA: Ribonucleic acid
RVFV: Rift Valley Fever Virus
S: Small
SNT: Serum Neutralization test
